# Identifying Amnestic Mild Cognitive Impairment With Convolutional Neural Network Adapted to the Spectral Entropy Heat Map of the Electroencephalogram

**DOI:** 10.3389/fnhum.2022.924222

**Published:** 2022-07-06

**Authors:** Xin Li, Yi Liu, Jiannan Kang, Yu Sun, Yonghong Xu, Yi Yuan, Ying Han, Ping Xie

**Affiliations:** ^1^Key Laboratory of Measurement Technology and Instrumentation of Hebei Province, Institute of Electric Engineering, Yanshan University, Qinhuangdao, China; ^2^College of Electronic and Information Engineering, Hebei University, Baoding, China; ^3^China-Japan Friendship Hospital, Beijing, China; ^4^Department of Neurology, Xuanwu Hospital of Capital Medical University, Beijing, China

**Keywords:** amnestic mild cognitive impairment, spectral entropy, convolutional neural network, early diagnosis, data augmentation

## Abstract

Mild cognitive impairment (MCI) is a preclinical stage of Alzheimer’s disease (AD), and early diagnosis and intervention may delay its deterioration. However, the electroencephalogram (EEG) differences between patients with amnestic mild cognitive impairment (aMCI) and healthy controls (HC) subjects are not as significant compared to those with AD. This study addresses this situation by proposing a computer-aided diagnostic method that also aims to improve model performance and assess the sensitive areas of the subject’s brain. The EEG data of 46 subjects (20HC/26aMCI) were enhanced with windowed moving segmentation and transformed from 1D temporal data to 2D spectral entropy images to measure the efficient information in the time-frequency domain from the point of view of information entropy; A novel convolution module is devised, which considerably reduces the number of model learning parameters and saves computing resources on the premise of ensuring diagnostic performance; One more thing, the cognitive diagnostic contribution of the corresponding channels in each brain region was measured by the weight coefficient of the input and convolution unit. Our results showed that when the segmental window overlap rate was increased from 0 to 75%, the corresponding generalization accuracy increased from 91.673 ± 0.9578% to 94.642 ± 0.4035%; Approximately 35% reduction in model learnable parameters by optimizing the network structure while maintaining accuracy; The top four channels were FP1, F7, T5, and F4, corresponding to the frontal and temporal lobes, in descending order of the mean value of the weight coefficients. This paper proposes a novel method based on spectral entropy image and convolutional neural network (CNN), which provides a new perspective for the identifying of aMCI based on EEG.

## Introduction

The preclinical stage of Alzheimer’s disease (AD) (the transition between normal aging and dementia) is termed mild cognitive impairment (MCI) (Arnáiz and Almkvist, 2003). MCI can present with a variety of symptoms, and when memory loss is the predominant symptom, it is termed amnestic mild cognitive impairment (aMCI) and is frequently seen as a prodromal stage of AD (Petersen et al., 2018). Therefore, the accurate diagnosis of patients with MCI and the corresponding intervention will help to delay the deterioration and even rehabilitation of patients with MCI. As a non-invasive detection method, electroencephalogram (EEG) detection is relatively mature [it has been widely used in clinical diagnosis, condition monitoring, brain-computer interface and other scenarios (Haas, 2003; Li et al., 2021; Soriano-Segura et al., 2021)]. It is more economical and practical than magnetic resonance imaging equipment in the purchase and maintenance of equipment, which makes it possible for low-cost large-scale screening of patients with cognitive impairment based on EEG analysis, especially in remote or underdeveloped areas. Moreover, a large number of studies have shown that EEG can be used as a carrier to represent cognitive level, patient status and other information or characteristics. Mizuno et al. (2010) pointed out that the characteristic of patients with AD lies not only in the decrease of the irregularity of EEG signals but also in the increase of entropy on a higher time scale. With the in-depth study of AD, several studies on spectral entropy have shown that spectral entropy can represent cognitive impairment information. Maturana-Candelas et al. (2019b) calculated the spectral entropy of EEG in 32 AD patients, 10 MCI subjects and 18 cognitively healthy controls (HC) on multiple scales. It was found that spectral entropy can be used as an effective pathological marker to characterize patients with cognitive impairment. Arakaki et al. (2019) found that the discrimination based on EEG spectral entropy was consistent with the detection of AD cerebrospinal fluid biomarkers, indicating that non-invasive EEG can be used as an important tool to distinguish between early dementia and normal aging. Compared with normal people, spectral entropy can describe the abnormalities of EEG signals in patients with cognitive impairment caused by functional loss caused by neuron death.

Spectral entropy measures the time-frequency domain metrics of the EEG signal in terms of information entropy, which provides more comprehensive information for the diagnosis of cognitive impairment and is therefore used to differentiate between MCI patients and normal subjects. Staudinger and Polikar (2011) found that the EEG signals of 161 subjects (79AD, 82HC) were less complex than those of the HC subjects through a non-linear metric and that reduced complexity could be attributed to the presence of neurofibril plaques and tangles. Specifically, the spectral entropy of the frontal lobe and temporal lobe in the AD group was also lower, and the electrodes F3, T7, and T8 had significant differences between the AD group and the HC group. Then, according to the combined feature vector, the multilayer perceptron (MLP) is used for pattern recognition, achieving an accuracy rate of 78%. Fan et al. (2018) made use of the spectral entropy characteristics of EEG signals of 123 participants (15HC, 108AD) on 20-time scales, and obtained about 80% test accuracy by regularization learning methods (Least absolute shrinkage and selection operator; Quadratic discriminant analysis). Ruiz-Gomez et al. (2018) used a non-linear feature measure including spectral entropy to classify 111 subjects (37AD, 37MCI, 37HC), with the MLP showing the highest diagnostic performance in determining whether the subjects were healthy or not (HC had a sensitivity of 82.35% for the ALL classification task and a positive predictive value of 84.85%). Sharma et al. (2019) analyzed 8 EEG biomarkers of 44 subjects (13HC, 16MCI, 15AD): power spectral density (PSD), skewness, kurtosis, spectral peak factor, spectral entropy, fractal dimension. Then it is classified by support vector machine (SVM), in which the classification accuracy of HC vs. MCI signal is 79.5%. Prior work is encouraging. These studies have demonstrated that non-linear metrics can adequately reflect the temporal, spatial and frequency characteristics of EEG. However, data redundancy in multi-channel EEG signals and the limited capabilities of traditional machine learning algorithms leave much to be desired in terms of the diagnostic performance of the models. Is there a way to better utilize this representational information to diagnose patients with cognitive impairment?

The answer is promising. Convolutional neural network (CNN) are widely used in the field of pattern recognition for their reliability, flexibility and ability to extract complex features from images. In this study, we focused on employing CNN to mine potential representational information in EEG signals from a spectral entropy perspective. The main contributions of this work are summarized below.

(1) The effectiveness of different window overlap rates in windowed moving segments on the enhancement of time series data was discussed.
(2) A novel convolutional model is constructed, which significantly reduces the number of parameters that can be learned by the model while ensuring diagnostic performance.
(3) One more thing, the contribution of the corresponding channels in each brain region can be measured by the absolute value of network weight.

## Materials and Methods

Firstly, the raw EEG signal is pre-processed, and the time series data is enhanced using a window shift segmentation method (with a window length of 4 s). The power spectrum and its spectral entropy were then calculated for each case to convert it into image data suitable for use as network input.

### Subjects and Electroencephalogram Recording

The data set used in this study is some of the clinical data reported by Su et al. (2021), including 26 subjects with aMCI with an average age of 65 years (60–70 years) and 20 HC subjects with an average age of 63.39 years (60–70 years). These aMCI patients were diagnosed by an experienced neurosurgeon and inclusion criteria included the Mini-Mental State Examination, Montreal Cognitive Assessment scores, and the Daily Living Test. In addition, subjects underwent MRI or CT to rule out focal lesions in the brain. All subjects had no history of other neurological disorders (e.g., depression, epilepsy, and brain injuries). Furthermore, MCI patients were not taking any neurological drugs during the EEG acquisition experiment.

The experiment was conducted following the Declaration of Helsinki and was reviewed and approved by the Medical Ethics Committee of Handan Central Hospital, Hebei Province, and all participants provided written informed consent. A digital electroencephalograph (NT9200, Beijing Zhongke Xintuo Instrument Co., Ltd., China) was used to record the resting-state EEG signals of 16 leads (frontal lobe FP1, FP2, F3, F4; left temporal lobe F7, T3, T5; parietal lobe C3, C4, P3, P4; right temporal lobe F8, T4, T6; occipital lobe O1, O2). The recording time of EEG of each subject was 15 min and the sampling rate was 1,000 Hz.

EEG preprocessing includes bandpass (0.5∼40 Hz) filtering and independent component analysis to remove the power frequency interference of 50 Hz, as well as ocular artifacts, electromyogram artifacts, abrupt slope and outliers. The preprocessed data set is segmented by a sliding time window with a window width of 4s and an overlap rate of 0, 25, 50, and 75% (the sliding steps are 4, 3, 2, and 1 s, respectively), and four enhanced data sets D00, D25, D50, and D75 are obtained. The specific description of the segmented data set is shown in Table 1, where each case is an EEG of 16 * 4,000 (channel * sample points).

**TABLE 1:** Description information of four segmented data sets.

Information category	D00	D25	D50	D75
Moving window size	4 s	4 s	4 s	4 s
Move window step (Overlap rate)	4 s (0%)	3 s (25%)	2 s (50%)	1 s (75%)
Number of cases in the HC group	3,433	4,579	6,874	13,758
Number of cases in the aMCI group	3,701	4,943	7,423	14,856
Total number of cases in the data set	7,134	9,522	14,297	28,614

### Electroencephalogram Data Transformation

Spectral entropy refers to the degree of uncertainty of the signal power spectrum distribution, which regards the normalized power distribution of the signal in the frequency domain as a probability distribution, and then calculates its Shannon information entropy. Specifically, for the EEG signal *x*(*n*), its discrete Fourier transform is *X*(ω), and the power spectrum is represented by *S*(ω) = |*X*(ω)|^2^. The probability distribution of the spectrum *p*(ω) is defined as:


$$p\,\left(\omega \right)=\frac{S\,\left(\omega \right)}{\sum_{i}S\,\left(i\right)}$$

Then the spectral entropy *H* is defined as:


$$H=-\sum_{\omega =1}^{N}\left(p\,\left(\omega \right)\,\log_{2}p\,\left(\omega \right)\right)$$

Here *N* is the total frequency point. In this paper, normalized spectral entropy is used, which is defined as:


$$H_{n}=-\frac{\sum_{\omega =1}^{N}\left(p\,\left(\omega \right)\,\log_{2}p\,\left(\omega \right)\right)}{\log_{2}N}$$

**FIGURE 1:**
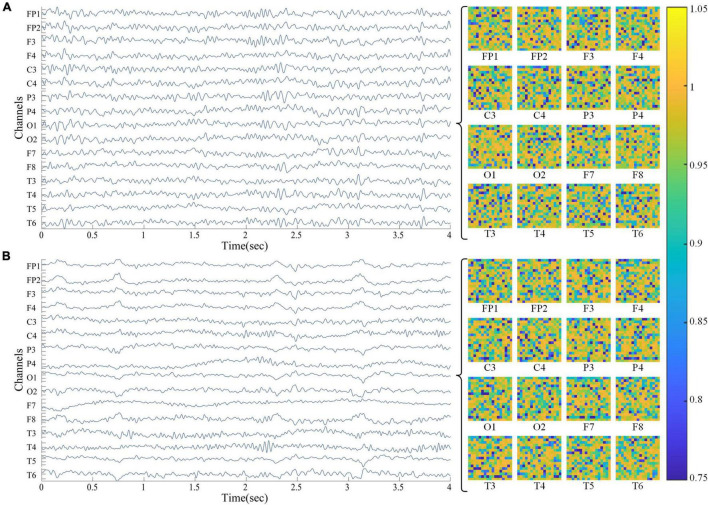
EEG and spectral entropy heat maps from subjects. **(A)** HC group; **(B)** aMCI group. The area on the left shows a 16-channel EEG, and the area on the right shows a 2-D spectral entropy image of the corresponding channel. In the spectrum entropy heat map, the abscissa is time and the ordinate is frequency bin.

Here the denominator *log*_2_⁡*N* represents the maximum spectral entropy in which the white noise is uniformly distributed in the frequency domain. The higher the spectral entropy of the signal, the more disordered (complex) the signal; on the contrary, the lower the spectral entropy, the more ordered (simple) the signal. As shown in Figure 1, 16 spectral entropy images (17*17) corresponding to the channel can be obtained from an EEG of 16 × 4,000 (channel * data points). In the right area of the image below, the Abscissa of the two-dimensional spectral entropy image is time, the ordinate is frequency bin, and the spectral entropy value is mapped to the color value in the thermal map.

In this study, the spectral entropy corresponding to each channel of the four datasets D00, D25, D50, and D75 was calculated in turn with a frequency resolution of 3 Hz. Correspondingly, each case in the feature image sets S00, S25, S50, and S75 consisted of 16 two-dimensional spectral entropy images corresponding to the channel. In addition, the power spectrum image set P75 of the dataset D75 with a window overlap of 75% as a control dataset for the spectral entropy dimensional conversion method.

### Classification Model

To mine the representation information in the spectral entropy image and identify the corresponding cognitive function patterns of the subjects, a CNN was constructed according to the characteristics of spectral entropy data sets S00, S25, S50, and S75. After the convolution operation of each layer, the spatial size of the output image data gradually decreases and the number of channels gradually increases. This work also follows this design principle when constructing the neural network model through Matlab, so as to avoid the information bottleneck. The main body of the base-model has a total of two convolution layers (Conv1, Conv2), followed by a full connection layer. Use the ReLU activation function to match the convolution layer, followed by the maximum pooling layer (Pool1, Pool2) to under-sample the image. After the convolution operation is completed, the full connection layer is connected, and the Softmax function is utilized as the final activation layer.

**FIGURE 2:**
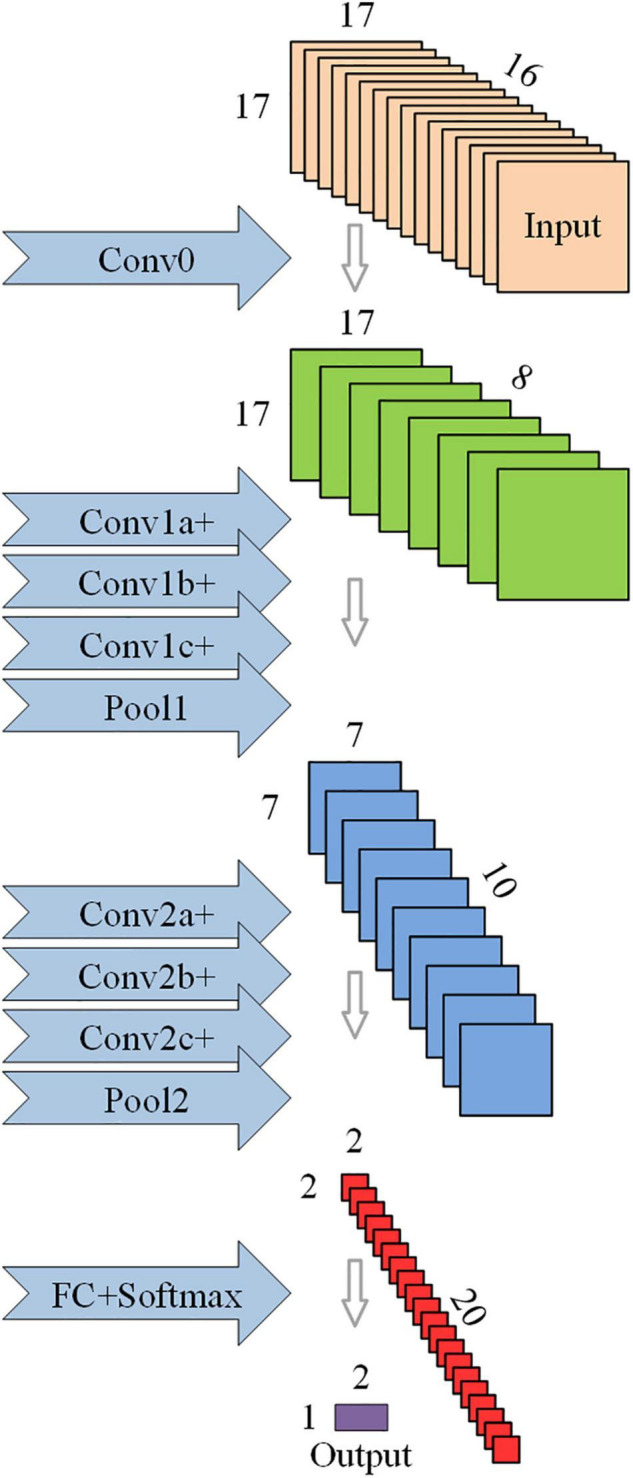
Schematic diagram of the structure of the optimized convolutional neural network (opt-model).

Inspired by the Inception Module (Szegedy et al., 2016), a novel network model architecture (opt-model) is constructed in the process of model debugging. Compared to the base model, the opt-model replaces the convolution layer (ConvN) with a convolution module (ConvNa, ConvNb, ConvNc). As shown in Figure 2, the convolution module has a parallel structure: one path is a series of two convolution layers to achieve a large convolution kernel, and the other uses the residual connection to achieve a smaller convolution kernel. Then the feature images are stacked by depth concatenation layer along the channel direction, and the linear combination of feature images between different channels is completed by the convolution layer (ConvNc, kernel-size = 1 × 1). Certainly, the convolution module is ingeniously designed. This structure can not only capture more comprehensive information from different scales through convolution kernels of different sizes but also reduce the total amount of learnable parameters and optimize the complexity of the model.

In addition, considering that the brain regions related to cognitive function in the cerebral cortex are the frontal lobe and temporal lobe (Al-Qazzaz et al., 2018; Zhang et al., 2021), a convolution layer (Conv0, kernel-size = 1 × 1) is immediately following the input layer to eliminate redundant data in EEG that is not related to the evaluation of cognitive function. This design can realize the linear combination of EEG data in different channels without greatly increasing the amount of calculation, instead of artificial selection, which can make the model more universal and more stable in the face of different subjects. Finally, the dropout layer (*P* = 0.95) is added in front of the full connection layer to prevent overmatching. Details of the base and opt model structure are given in Table 2, containing the parameters of some of the layers and their corresponding output sizes, and the number of learnable parameters.

**TABLE 2:** Network model parameters corresponding to Figure 2.

Layer_base_	Num, size	Output size	Σ*N*_*learnable parameter*_	Layer_opt_	Num, size	Output size	Σ*N*_*learnable parameter*_
I	–	[17, 17, 16]	–	I	–	[17, 17, 16]	–
–	–	–	–	Conv0	8, [1, 1]	[17, 17, 8]	136
Conv1	10, [5, 5]	[13, 13, 10]	4,010	Conv1a	8, [3, 3]	[15, 15, 8]	584
–	–	–	–	Conv1b	8, [3, 3]	[15, 15, 8]	584
–	–	–	–	Conv1c	10, [1, 1]	[15, 15, 16]	170
Conv2	20, [3, 3]	[4, 4, 20]	1,820	Conv2a	10, [3, 3]	[5, 5, 10]	910
–	–	–	–	Conv2b	10, [3, 3]	[5, 5, 10]	910
–	–	–	–	Conv2c	20, [1, 1]	[5, 5, 20]	420
F	2	[1, 1, 2]	162	F	2	[1, 1, 2]	162

For the multi-channel signal represented by EEG, the contribution of each EEG channel can be measured by the absolute value of the weight coefficient of the input unit and the hidden layer unit. Specifically, when the absolute value of the weight tends to be 0, the smaller the importance of the channel, the smaller the contribution of the channel to the representation of the patient’s cognitive state. In this study, the weight parameters of the convolution kernel (kernel-size = 1 × 1) in the convolution layer Conv0 of Figure 2 were used to evaluate the contribution of EEG 16 channels and their corresponding brain regions. The algorithm of channel contribution evaluation based on convolution kernel weights is given in Algorithm 1, where *W_k,m,n_* represents the weight of the *m-th* convolution kernel in the channel *n-th* of the target convolution layer of the corresponding model in the *k-th* fold cross-validation.

**Algorithm 1:** Evaluation of channel importance algorithm by convolution kernel weight.

**Input:** The CNN model is obtained by K-fold cross-validation, in which the total number of channels to be evaluated is N.
**Output:** Evaluation value (*P_n_*) of channel importance.
1: Obtain the weight parameter *W_k,m,n_* of all convolution kernels (convolution kernel size = 1 × 1) in the target convolution layer of the CNN model.
2: **while *n* ≤ *N* do**
3: $M_{n}\leftarrow \left(\sum_{m}\sum_{K}\left|W_{K,m,n}\right|\right)/\left(m+K\right)$
4: $S_{n}\leftarrow \sqrt{\left\{\sum_{m}\sum_{K}\left({|W_{K,m,n}-M_{n}|}^{2}\right)\right\}/\left(m+K-1\right)}$
5: $P_{n}\leftarrow M_{n}*S_{n}$
6: *n++*
7: **end while**
8: **return** *P_n_*.


The objective function of the two convolution models is the cross-entropy loss function. Initialize the weights with the Glorot initializer (also known as Xavier initializer) (Glorot and Bengio, 2010), and initialize the bias with the zeros. Because of the large amount of calculation, the model training uses the Adam optimizer with high computational efficiency (Kingma and Ba, 2015), in which *β1* is set to 0.9, *β2* is set to 0.999, and the learning rate is set to 10^(–5)∼10^(–4). The number of training epoch is 100 and the batch size is 200. These super-parameters are determined by grid optimization and artificial testing, and the optimized model provides the best results in terms of accuracy, loss and generalization performance.

### Evaluation Metrics

In the field of pattern recognition, the performance evaluation of the model is essential. K-fold cross-validation is a statistics-based evaluation method to show the expected performance on unknown cases. In this paper, a 10-fold cross-validation method is adopted. Specifically, the spectral entropy dataset S00 was first randomly shuffled and folded to reduce the effect of data order on the model. For each fold, 9/10 fold for training (Among them, 8.1/10-fold is used to learn and update network weights, and 0.9/10 fold discount is used to verify and prevent model overfitting), and 1/10 fold for testing and evaluation of models. Secondly, the performance of the model corresponding to this dataset is evaluated by the average values of accuracy, precision, specificity, sensitivity (also known as Recall), F1-Score, Area Under ROC Curve (AUC) and average validation accuracy curve, average verification loss curve and receiver operating characteristic curve (ROC). The models were then trained by datasets S25, S50, and S75 in turn, which resulted in model performance being accessed for the corresponding four datasets.

### Statistical Analysis

A two-sample Kolmogorov-Smirnov test was used to check the spectral entropy data between aMCI and HC and whether there were significant differences in model performance corresponding to different segmental overlap rates.

## Results

This paper focuses on the effectiveness of the spectral entropy image conversion method, the influence of different overlap rates in time series signal segmentation on the performance of the CNN model, and the contribution of verifying each brain region in the assessment of cognitive impairment. Specifically, on the basis of preprocessing and dimensionality conversion of all subjects’ EEG signals, spectral entropy image data sets S00, S25, S50, S75 were obtained, in which the aMCI group was marked as 1, the HC group was marked as 0. After analyzing the influence of data segment overlap rate on the performance of the model by base-model, the accuracy and complexity of the model before and after optimization and the richness of the spectral entropy image conversion method investigated to the conventional time-frequency representation are compared and analyzed. According to the structural advantages of the opt-model, the contribution of corresponding channels in each brain region for the evaluation of cognitive impairment is discussed.

### The Influence of Different Segmentation Overlap Rate on Model Training

This section shows the performance evaluation of CNN classification corresponding to four different segment overlap rates, compares and analyzes the corresponding results, and discusses the effectiveness of the segmentation strategy as a time series data enhancement method.

**FIGURE 3:**
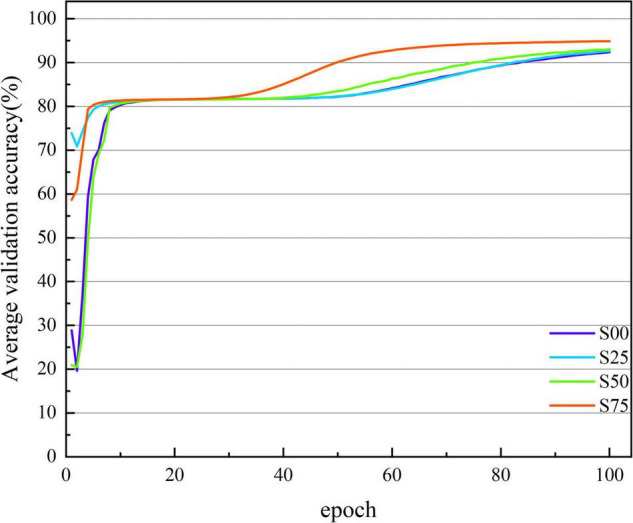
The average verification accuracy curves of the base-model under different segmentation overlap rates. The abscissa is the number of epochs, whose range of values is from 1 to 100; and the ordinate is the average verification accuracy rate, whose range of values is from 19.631 to 94.888%.

**FIGURE 4:**
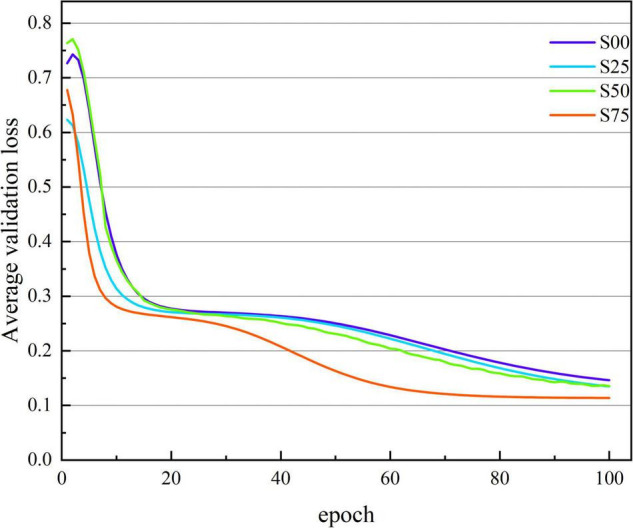
The average verification loss curve under different segmentation overlap rates. The abscissa is the number of iterations, whose range of values is from 1 to 100; and the ordinate is the average verification loss rate, whose range of values is from 0.771 to 0.114.

Figures 3, 4 show the average accuracy and loss curves of the first 100 epochs of base-model on the corresponding validation data set, respectively. During the training period, the average 10-fold verification accuracy of the model increases steadily with the increase of epoch, and the verification loss decreases steadily with the increase of the number of iterations. When the epoch reaches 90–100 times, the model is saturated, that is, the training error and verification error are the same. At this time, the average verification accuracy corresponding to dataset S00 is maintained at 92.371 ± 1.226%, and the average verification loss is maintained at 0.146 ± 0.019; the average verification accuracy corresponding to dataset S25 is maintained at 92.643 ± 1.236%, and the average verification loss is maintained at 0.135 ± 0.017; the average verification accuracy corresponding to dataset S50 is maintained at 93.042 ± 0.641%, and the average verification loss is maintained at 0.135 ± 0.013; the average verification accuracy corresponding to dataset S75 is maintained at 94.888 ± 0.483%, and the average verification loss is maintained at 0.114 ± 0.016. If we continue the training, the verification error shows an upward trend and exceeds the training error, there is no doubt that the model is overfitted at this time. In order to avoid this phenomenon, the network training, namely early stopping, is stopped when the accuracy of the verification data is not improved 10 times in a row.

**FIGURE 5:**
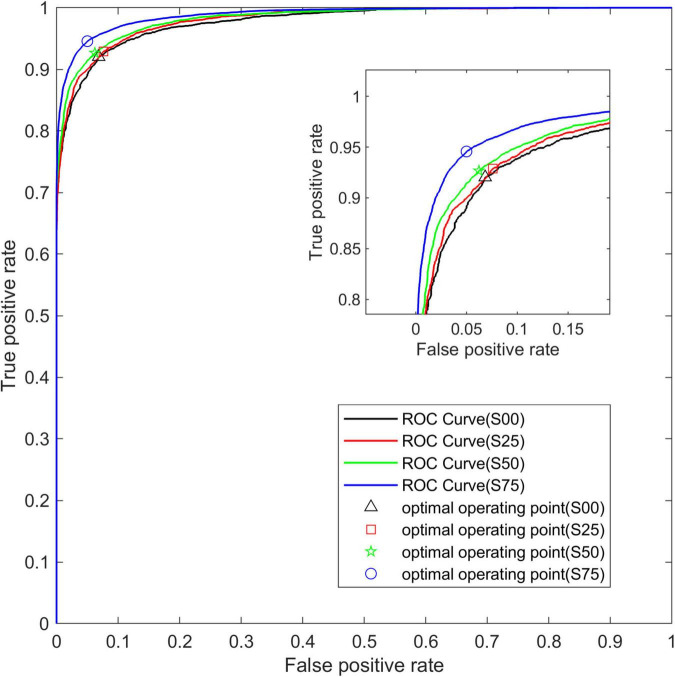
The average ROC curve under different segmentation overlap rates. The abscissa is False Positive Rate (1-Specificity) and the ordinate is True Positive Rate (Sensitivity).

Figure 5 shows the average ROC curve on the test set for the four segmentation strategies. The ROC curve obtained from the data set S75 is closer to the upper left corner than the other three ROC curves, and the area under the ROC curve, namely AUC, increases with the increase of overlap.

**FIGURE 6:**
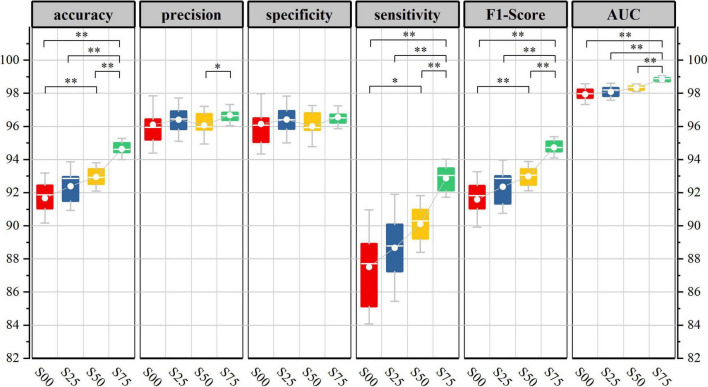
Base-model test performance corresponds to different segmentation overlap rates. (10-fold cross-validation corresponds to model performance metrics in the test set, Kolmogorov-Smirnov test, *p < 0.05, ^**^p < 0.01).

Figure 6 shows the generalization performance metrics of the base-model on test datasets corresponding to four-segment overlap rates. In the testing period, with the increase of segment overlap rate, the 10-fold cross-validation mean of each evaluation index value of the base-model has been improved in varying degrees, and the corresponding low standard deviation also shows the robustness and consistency of the model.

According to the performance of the same model in the data set corresponding to the overlapping rate of different segments, it can be found that windowed segmentation is an effective data enhancement method for small sequential data sets. When constructing the pattern recognition model represented by EEG, sample size and calculation amount should be considered in equilibrium, and the overlap rate can be selected from 50 to 75%.

### Performance Comparison Pre and Post Model Optimization

In view of the fact that the windowed moving segmentation method with an overlap ratio of 75% is more likely to get a model with good performance, it is used to train the opt-model. Based on datasets S75 and P75, this section compares and analyses the performance differences of the models before and after optimization, and the richness of the spectral entropy image conversion method compared to the power spectrum.

**FIGURE 7:**
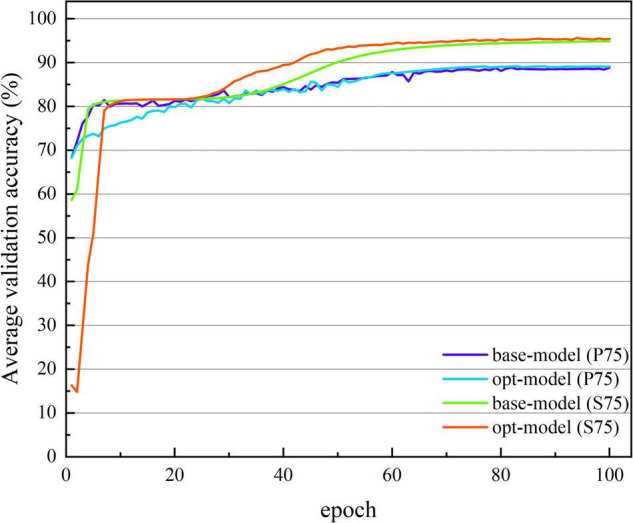
The average verification accuracy curve of two network structures. The abscissa is the number of iterations, whose range of values is from 1 to 100; and the ordinate is the average verification accuracy rate, whose range of values is from 14.789 to 95.517%.

**FIGURE 8:**
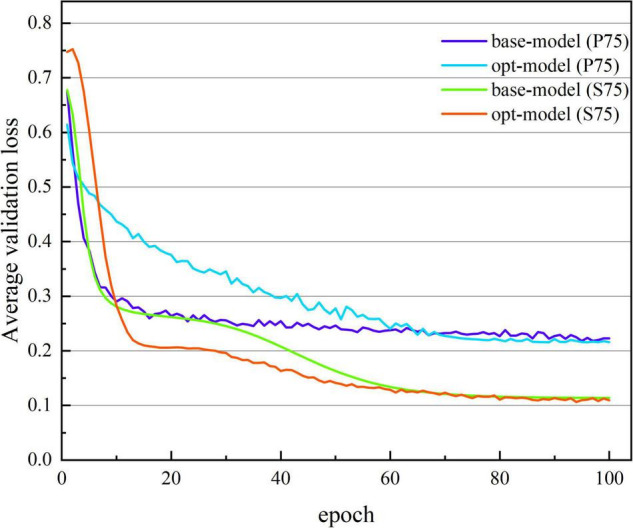
The average verification loss curve of two network structures. The abscissa is the number of iterations, whose range of values is from 1 to 100; and the ordinate is the average verification loss rate, whose range of values is from 0.752 to 0.108.

Figures 7, 8 show the average verification accuracy and loss curves of base-model and opt-model network models in P75 and S75. For S75: at the beginning of training, the average verification accuracy of the opt-model is much lower than that of the base-model, and the average verification loss is higher than the base-model. However, with many iterations of the model, the accuracy of the opt-model improves rapidly, and the verification loss of the 10th epoch is lower than that of the base-model. When training proceeds to the 60th stage, the average validation accuracy and loss curves of the two models smooth out and no gross fluctuations occur during the subsequent iterations. When the 100th epoch is trained, the average verification accuracy of the base-model is maintained at 94.888 ± 0.483%, and the average verification loss is maintained at 0.114 ± 0.013; the average verification accuracy of the opt-model is maintained at 95.414 ± 0.791%, and the average verification loss is maintained at 0.110 ± 0.016.

For P25: the performance of the opt-model varied less than that of the base-model at the beginning of the training, but the difference between the two models was not significant as the model was iterated. By the 100th epoch, the average validation accuracy of the base-model remained between 89.093 ± 2.536% and the average validation loss remained at 0.216 ± 0.054; the average validation accuracy of the opt-model remained at 88.859 ± 3.433% and the average validation loss remained at 0.223 ± 0.061. Overall, the model obtained from spectral entropy outperformed the power spectrum, and the difference became apparent as the training progressed to 40 epochs.

**FIGURE 9:**
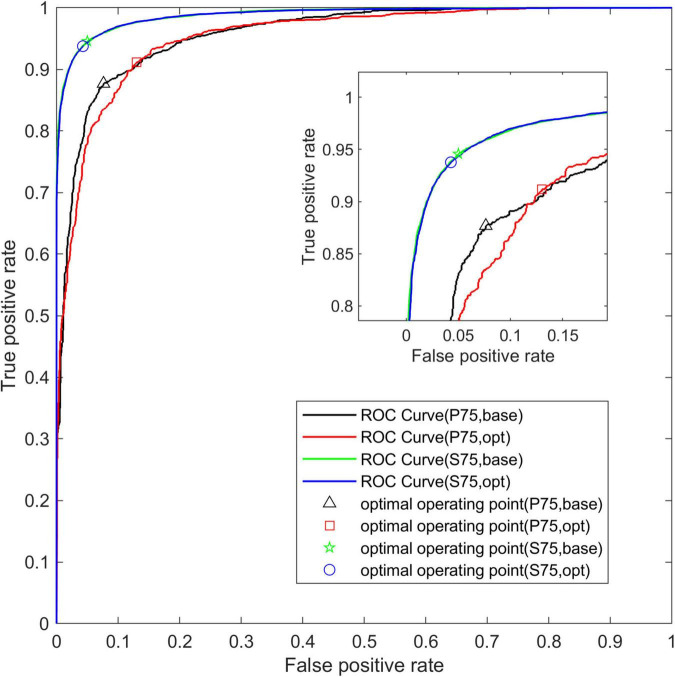
The average ROC curve of two network structures. The abscissa is False Positive Rate (1-Specificity) and the ordinate is True Positive Rate (Sensitivity).

Figure 9 shows the average ROC curve of the base-model and the opt-model on the P75 and S75 test sets. Through the ROC curve, we can see that the two model structures have better comprehensive performance in the data set.

**FIGURE 10:**
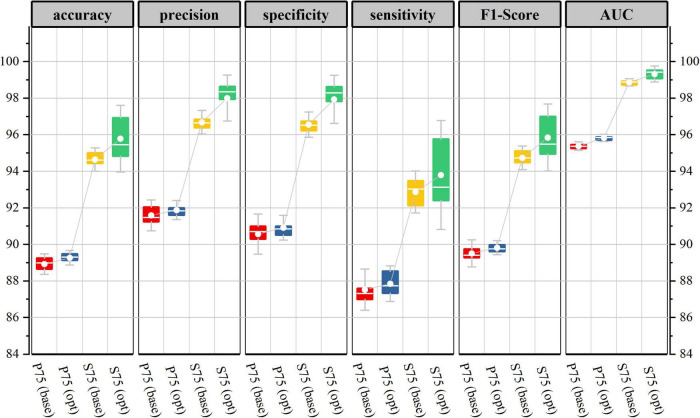
Test performance of two network structures on data sets P75, S75.

Figure 10 shows the performance evaluation metrics of the two models on the test set of the P75 and S75. It can be seen that during the testing period, the generalization performance of the two models is almost the same. The total number of learnable parameters of the base-model is 5992, which has the eye-catching characteristics of simple architecture, but it is at the cost of high computational cost: network training and evaluation require a lot of computation. In contrast, the total number of learnable parameters of the opt-model is 3876, which can be performed well even under strict memory and computing budget constraints.

Thus, spectral entropy may contain additional information to indicate the state of cognitive impairment compared to the traditional time-frequency representation of the power spectrum. In addition, there is a great difference in the number of learnable parameters caused by the structure (the total number of opt-model learnable parameters is reduced by about 35.31%), which shows that the convolution module designed in this paper can effectively reduce the number of learnable parameters while maintaining high performance.

### Contribution Evaluation of Electroencephalogram Channel Based on Convolution Kernel Weight

**FIGURE 11:**
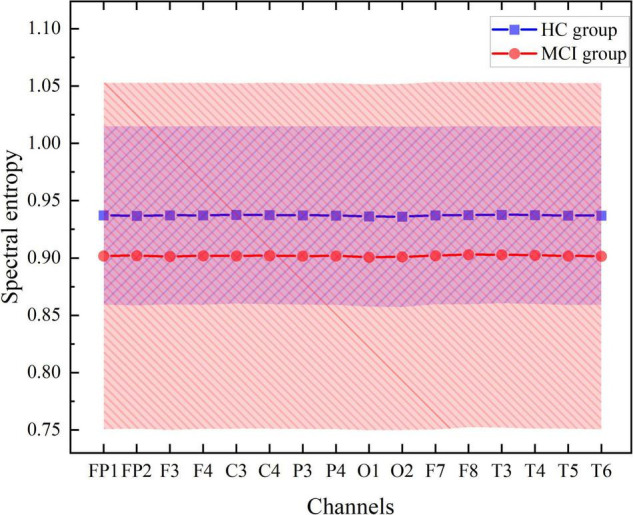
Spectral entropy values corresponding to 16 channels.

This section shows the contribution of each EEG channel and its corresponding brain regions measured by the absolute weight of the convolution kernel. Firstly, the 16-channel spectral entropy of all subjects in aMCI group and HC group were tested by double-sample Kolmogorov-Smirnov test, and it was found that there were significant differences in spectral entropy data of all channels (*P* = 0.01). The fluctuations in spectral entropy data corresponding to the 16 EEG channels are conveyed by Figure 11, which shows similar patterns of variation in spectral entropy data for each channel within the group, with significant differences between groups.

**FIGURE 12:**
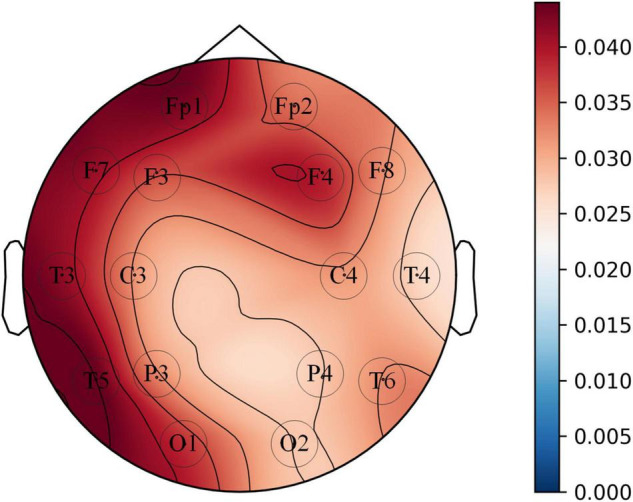
Topographic map of the average value of 8 convolution kernel (kernel-size = 1 × 1) weights corresponding to EEG signal channels.

The diagnostic contribution of the corresponding channels in each brain region is illuminated in Figure 12. The contribution assessment topography is quantified by the convolutional layer (Conv0) weights used to reduce the number of channels in the opt-model, and the drawing data derived from the channel contribution assessment values returned from Algorithm 1. The contribution evaluation values (P_*n*_) of the 16 channels are sorted in the following order: FP1 > F7 > T5 > F4 > C4 > F3 > T3 > FP2 > O1 > T6 > C3 > P3 > F8 > O2 > T4 > P4. Two of these brain regions, the frontal lobe (FP1, FP2, F3, F4) and the left temporal lobe (F7, T3, T5), have relatively higher weighting coefficients. The frontal, temporal lobes and the hippocampus medial to the temporal lobe, are associated with auditory and language expression, processing, and memory functions. Correspondingly, The language impairment, executive impairment, cognitive impairment and memory impairment experienced by the aMCI subjects in this paper may be related to damage to these brain regions.

At the same time, the average absolute weight of all channels is not close to zero, that is, all channels have an indelible contribution to the assessment of cognitive impairment. This can be explained that the normal expression of cognitive function is a manifestation of the brain’s comprehensive ability, and no brain region can be left alone; and each brain region may dominate a certain cognitive function. Different patients may be involved in different brain regions when their cognitive function is impaired. Therefore, in the actual clinical diagnosis, we need to comprehensively consider each brain region, which can avoid misdiagnosis and missed diagnosis to a certain extent.

## Discussion

### Feasible and Effective of Spectral Entropy Methods

Since the recorded EEG is a combination of external electrical pulse activity and synaptic signals of cortical neurons, some scholars (Nunes et al., 2004) have indicated that spectral entropy is not only a statistical measurement of the range/degree of variability or regularity and disorder of EEG signal patterns but also accurately reflects cortical function and “intracortical information flow.” From a nervous system point of view, EEG complexity or irregularity is related to the dynamic complexity of part of the brain, as well as the lack of neurotransmitters, neuron death, and even changes in network structure. Spectral entropy may be more likely to associate entropy with the number of current cortical microstates of the brain than to measure the degree of disorder of the brain process. Therefore, the signal spectral entropy that describes the energy of the brain is more likely to represent the number of frequency components, for example, the higher the spectral entropy may represent the more the number of mental microstates that the brain exists in that particular period.

Abasolo et al. (2006) found that the approximate entropy in the parietal region and the sample entropy in the parietal and occipital regions of AD patients decreased significantly. Al-Qazzaz et al. (2016) by analyzing the spectral entropy and relative power of five scalp regions in patients with MCI and age-matched control subjects, it was found that the spectral entropy in the parietal, occipital and central regions in the MCI group was significantly lower than that in the control group, and the difference was statistically significant (*p* < 0.05). Zhang et al. (2021) statistically analyzed the scores of two neurological tests (MMSE and MoCA) and resting-state EEG in 30 normal elderly subjects and 30 patients with AD. It was found that in the α band, the spectral entropy of the frontal, temporal and central regions in the AD group was significantly lower than that in the HC group, but there was no significant difference in the spectral entropy of the occipital lobe electrode between the two groups; For β band, the spectral entropy of temporal region, central region and occipital region in the AD group was significantly higher than that in HC group, but there was no significant difference in the frontal region between groups; The spectral entropy of θ band in the occipital region of AD group was higher than that of HC group. In this study, a two-sample Kolmogorov-Smirnov test analysis of the subjects’ EEG spectral entropy eigenvalues revealed that the aMCI group was significantly lower than the HC group and that the frontal and left temporal lobes may have critical brain regions in characterizing cognitive state information, which is consistent with the findings of the above study.

Taken together, these studies have shown that EEG can be used as an effective detection tool for the preclinical stage of AD. At the same time, the results of this study further prove that the spectral entropy is worth considering, and the non-linear measure can mine the potential representation information in the EEG spectrum, so it is reasonable and effective to apply the spectral entropy image to distinguish the aMCI patients and the control group. In addition, according to the value of the opt-model’s convolution layer (Conv0) weight parameter, it is found that the contribution of 16 channels to classification performance is in the same order of magnitude. It may be that the sound expression of cognitive function needs the cooperation of five brain regions, so when designing the medical assistant diagnosis system, in order to accurately and stably complete the early evaluation of patients with cognitive impairment, diagnostic modeling should be combined with the EEG signals of all brain regions.

### The Clinical Performance of the Opt-Model

So far, many scholars have carried out other related studies to address the issue of pattern classification of cognitive impairment. Compared with previous spectral entropy correlation studies (Staudinger and Polikar, 2011; Wang et al., 2015; Al-Qazzaz et al., 2017, 2018; Ruiz-Gomez et al., 2018; Maturana-Candelas et al., 2019a; Sharma et al., 2019), this paper creatively combines spectral entropy theory with Deep learning (DL) and achieves better classification performance. Figures 3, 7 show that with the increase of the number of iterations, the model gradually fits the data samples and achieves better accuracy. Figures 4, 8, respectively, illuminate the verification loss, which not only reflects the excellent classification performance of the scheme but also shows that its convergence is relatively stable. And when the segmentation window overlap rate increases from 0 to 75%, the corresponding generalization accuracy increases from 91.673 ± 0.9578% to 94.642 ± 0.4035%. Then the structure of the model is optimized, and the generalization accuracy of the opt-model is 94.586 ± 0.4224% when the total number of learnable parameters is reduced by about 35.31%. Compared with the research (Morabito et al., 2016; Ieracitano et al., 2019, 2020; Wen et al., 2020; Huggins et al., 2021) related to CNN, the model performance of this paper is second only to the study by Huggins et al. (2021). But the diagnosis accuracy is on the same order of magnitude, it benefits from the irregularity and complexity representation information in the spectral entropy image. In contrast to transfer learning, this paper aims to artfully conceive of a model structure that allows for a significant reduction in parameters and savings in computational resources. It is thus reasonable to trade off model performance in a comprehensive manner in practical applications.

In the future, more aMCI/HC subjects will be recruited to prevent their data samples from being included in the testing phase and affect the objectivity of the diagnostic model assessment. In addition, low-density EEG signals from 16 electrodes were used in this study. A larger number of EEG channels can provide a more comprehensive and stable picture of the subjects’ cognitive function, thus improving classification performance. For this reason, subsequent attempts will be made to record high-density EEG recordings and to introduce individual information (age, weight, height, etc.) into the diagnostic protocol to enhance the robust performance of the model. Furthermore, as a type of physiological electrical signal, some of the results of this study can be used not only in the design and development of cognitive assessment systems, but also in the application of other physiological signals such as EMG and ECG. In the meantime, our study is an exploratory study and requires more data samples and subsequent studies for validation.

## Conclusion

A novel method for the detection of aMCI based on EEG time-frequency analysis was proposed. That is, after the time-frequency analysis of the EEG signal with the spectral entropy, CNN is used to extract the potential information in the spectral entropy images. The performance of the method with different overlap window overlap rates is given in the paper. The results show that for a 75% segmented window overlap rate, the highest accuracy can reach 95.077%. In addition, to reduce the complexity of the model, a convolution module is proposed, which reduces the learnable parameters of the model by about 35% while maintaining accuracy (Total number of opt-model learnable parameters: 3,876). One more thing, the research results of this paper provide a new way of thinking on how can effectively use CNN to mine the potential representation information in EEG and identify aMCI abnormalities. This facilitates large-scale screening and cognitive ability testing of patients with cognitive impairment; It also contributes to the development of remote diagnostic systems for neurological diseases, which are necessary for remote towns and villages that lack trained neurologists and facilities.

## Data Availability Statement

The raw data supporting the conclusions of this article will be made available by the authors, without undue reservation.

## Ethics Statement

The studies involving human participants were reviewed and approved by the Medical Ethics Committee of Handan Central Hospital, Hebei Province. The patients/participants provided their written informed consent to participate in this study.

## Author Contributions

XL provided reviewing, editing, and supervision. YL proposed the idea, finished the neural model coding, and wrote the manuscript. YH and JK offered important help on EEG processing and analysis methods. YS, YX, and YY provided advice on the research approaches, guided the experiments, and checked and revised the manuscript. PX verified the repeatability of the results of the network experiment. All authors revised the manuscript and approved the submitted version.

## Conflict of Interest

The authors declare that the research was conducted in the absence of any commercial or financial relationships that could be construed as a potential conflict of interest.

## Publisher’s Note

All claims expressed in this article are solely those of the authors and do not necessarily represent those of their affiliated organizations, or those of the publisher, the editors and the reviewers. Any product that may be evaluated in this article, or claim that may be made by its manufacturer, is not guaranteed or endorsed by the publisher.
